# The evolution of antibody-secreting cell biological exploration: one mouse model at a time

**DOI:** 10.3389/fimmu.2026.1739007

**Published:** 2026-02-06

**Authors:** KimAnh Trang Pioli, Peter Dion Pioli

**Affiliations:** Department of Biochemistry, Microbiology and Immunology, College of Medicine, University of Saskatchewan, Saskatoon, SK, Canada

**Keywords:** antibody-secreting cell, CD138, Jchain, mouse model, plasma cell, plasmablast, PRDM1, timestamp

## Abstract

Upon antigenic exposure, B cells become activated and undergo differentiation into antibody-secreting cells (ASCs) which secrete thousands of antibodies per second. However, ASCs have shown themselves to be a heterogenous population which possesses capabilities well beyond just antibody secretion. Therefore, a growing need exists to more comprehensively assess ASC biology. As the rise of genetically modified mouse models has allowed for substantial gains in our understanding of immune system development and function, it also enables the exploration of ASC development and function. This Perspective article aims to highlight the evolution in mouse models which have facilitated the advancement of ASC research in areas including development, function, heterogeneity and longevity. Furthermore, we discuss the future of ASC mouse models as it pertains to their potential of furthering our understanding of ASC biology in health and disease.

## 1 Introduction

Humoral, or antibody-mediated, immunity plays a critical role in not only the response to pathogenic infection, but also in prevention of infection. The latter is driven largely by the presence of long-term immunological memory that is present upon re-exposure to a pathogen. Related to this, antibody-secreting cells (ASCs) are terminally differentiated B cells which arise following B cell activation. As their name suggests, ASCs are professional antibody factories due to transcriptional programming that allows for an expanded endoplasmic reticulum and Golgi apparatus (1). Early evidence of the long-term antibody response was provided using mouse models of lymphocytic choriomeningitis virus (LCMV) infection (2, 3). This work revealed that ASCs in the bone marrow provided a durable source of LCMV-specific antibodies lasting over a year following infection whereas numbers of LCMV-specific ASCs in the spleen waned within ~2 months (3). However, it was still not clear whether the ASCs themselves had long-lived potential or if their presence was a result of continuous memory B cell differentiation. The subsequent experiments utilizing strategies to deplete memory B cells as well as adoptive transfer of ASCs provided clear evidence of the long-lived potential of LCMV-specific ASCs (2). Complementing these observations, Manz et al. (4) utilized the model antigen ovalbumin (OVA) to demonstrate the ability of adoptively transferred ASCs to sustain long-term antibody responses in the absence of antigen. Furthermore, these studies indicated that this activity was largely confined to a fraction of ASCs lacking B220 expression and that OVA-specific ASCs downregulated major histocompatibility complex class II (MHC II) expression over time (4). Essentially, these were some of the earliest experiments to demonstrate ASC heterogeneity and maturation. Since then, it has been shown that mouse ASCs which express high levels of CD138 (Syndecan-1) can be subdivided into short-lived, proliferative B220^+^ plasmablasts and mature B220^-^ plasma cells (5). Within the B220^-^ plasma cell compartment, the presence or absence of CD19 can further subdivide cells (5–7) with CD19^-^ surface phenotype being correlated with a mature resting phenotype (5, 7).

Not surprisingly, B cell to ASC differentiation is driven by coordinated networks of gene expression (8–10) such that upregulation of factors including *Irf4*, *Prdm1* (BLIMP-1), *Sdc1* (Syndecan-1), *Xbp1* and *Jchain* has been associated with an ASC fate. In contrast, ASC differentiation normally correlates with downregulation of B cell and germinal center-related genes such as *Cd19*, *Pax5* and *Bcl6* (8–10). Further complicating these transcriptional changes is the cross regulation that occurs between gene networks. For instance, PAX5 and BCL-6 (11) suppress expression of *Prdm1* whereas BLIMP-1 blocks activation of *Pax5* and *Bcl6* genes (12, 13). While extensive progress has been made in the field of ASC biology especially with the advent of single cell genomics, much remains to be uncovered especially regarding aspects of cellular heterogeneity, longevity and function in both health and disease. To that end, various mouse models that are largely specific to ASC biology have been constructed. Here, we aim to highlight some of the key mouse models generated over time that have contributed and will continue contributing to the study of ASCs.

## 2 Constitutive fluorescent reporters for ASC identification

BLIMP-1, encoded by *Prdm1*, is a ~98 kilodalton zinc finger DNA binding protein that was originally shown by Turner et al. (14) in 1994 to be rapidly upregulated following interleukin (IL)-2 and IL-5 induced differentiation of BCL1 cells into ASC-like cells. Furthermore, this group demonstrated that overexpression of BLIMP-1 in BCL1 cells could induce CD138 and *Jchain* expression in conjunction with IgM secretion (14). These results led to subsequent studies in 2002 by Shaffer et al. (13) indicating that ectopic BLIMP-1 expression in B cell lines and in activated splenic B cells could lead to genome transcriptional changes and suppress B cell identify genes such as *Pax5*, *Ebf*, *Spib* and *Bcl6* among others. In 2003, Shapiro-Shelef et al. (15) generated a mouse line with a conditionally deletable floxed *Prdm1* allele which was bred to *Cd19*-Cre expressing mice resulting in *Prdm1* deletion within the B cell lineage. Examination of these mice provided *in vivo* evidence that *Prdm1* (BLIMP-1) acted as a master transcription factor which was essential for ASC differentiation (15). This was demonstrated by a loss of serum IgM, IgA and multiple IgG isotypes in naïve mice lacking *Prdm1*. Additionally, antibody responses to immunization with T cell-independent and T cell-dependent antigens were defective demonstrating the universal requirement of *Prdm1* in ASC formation (15).

The realization that BLIMP-1 acted as a master transcription factor for ASC development led to the development of mouse models that possessed fluorescent reporter genes under the control of *Prdm1* thus providing a convenient means of ASC identification using flow cytometry (**Table 1**). Not only was this strategy technically straightforward from an analytical perspective but it was essential as many other “now accepted” ASC markers had yet to be identified and demonstrated to be reliable [e.g., CD93 (16), CD98 (10), CD267(TACI) (7)]. One of the first examples of the above approach was the development of *Prdm1*-green fluorescent protein (GFP) mice by Kallies et al. in 2004 (17). In this instance, GFP was inserted downstream of exon 6 of the *Prdm1* gene creating both a reporter that reflected *in vivo* gene expression but also a BLIMP-1 loss of function truncation. Since homozygous expression of this reporter was embryonic lethal, the authors relied upon the use of *Prdm1*^+/GFP^ heterozygous animals to demonstrate that BLIMP-1 levels positively correlated with ASC maturation (17). Indeed, GFP^HI^ (i.e., BLIMP-1^HI^) ASCs expressed lower levels of CD19, B220 and MHC II compared to GFP^INT^ ASCs whereas the GFP^INT^ cells displayed increased BrdU labeling which was associated with a plasmablast phenotype (17). In 2007, Kallies et al. (18) used these same reporter mice to transplant fetal liver from *Prdm1*^+/GFP^ and *Prdm1*^GFP/GFP^ into *Rag1*^-/-^ mice to assess *Prdm1* dependency for ASC formation. Similar to *Cd19*-Cre deletion of *Prdm1* (15), serum harvested from mice transplanted with *Prdm1*^GFP/GFP^ fetal liver cells showed a reduction in antibody levels although this phenotype was far from absolute. These experiments further suggested that initiation of ASC development did not necessarily require BLIMP-1 (18); however, the interpretation of these results is complicated as the *Prdm1*-GFP allele still makes a truncated BLIMP-1 protein which may retain a low level of activity (17). Proximal to the above study, Fairfax et al. (19) isolated various B cell subsets from *Prdm1*^+/GFP^ mice to determine differences in ASC differentiation potential following stimulation. Using GFP expression as a surrogate for BLIMP-1 expression, they showed quantitative differences regarding the kinetics of BLIMP-1 upregulation with B-1 B cells being the most rapid followed by marginal zone B cells then B-2/follicular B cells (19). Moreover, adoptive transfer experiments demonstrated the ability of both B-1 and B-2 B cells to reconstitute the ASC compartment in the bone marrow (19). These results as well as others have led to multiple studies examining the differential contributions of B cell subsets to ASC generation (20, 21).

**Table 1:** List and brief description of *Prdm1* fluorescent reporter models.

Mouse model	Year	Authors	Brief description
*Prdm1*-GFP (*Prdm1*^+/GFP^, *Prdm1*^GFP/GFP^)	2004	Kallies et al. (17)	GFP inserted downstream of *Prdm1* exon 6 and BLIMP-1 (i.e., GFP) levels were correlated with ASC maturity.
	2007	Kallies et al. (18)	Demonstrated ASC dependency on *Prdm1* using fetal liver transplantation into *Rag1*^-/-^ mice.
	2007	Fairfax et al. (19)	*In vitro* differentiation of B cell subsets into ASCs.
*Prdm1*-YFP	2017	Fooksman et al. (28)	YFP inserted into *Prdm1* exon 2 on a bacterial artificial chromosome and used to show ASC regulation by myeloid populations.
	2019	Rojas et al. (34)	IL-10-producing gut IgA ASCs regulated neuroinflammation.
	2020	Fitzpatrick et al. (36)	Imaged ASCs in the meninges and examined clonal relatedness of meningeal and gut ASCs.
	2023	Pioli et al. (6)	Identified thymic ASCs and profiled gene expression using single cell RNA-sequencing.
	2024	Pecha et al. (35)	Showed that common enzymatic tissue digestion protocols destroyed CD138 surface expression.

While the Kallies et al. (17) *Prdm1*-GFP model has been widely utilized to study aspects of ASC biology (5, 22–27), a complication is that this construct ablates endogenous BLIMP-1 function thus eliminating the ability to maintain *Prdm1*^GFP/GFP^ mouse lines. In 2014, Fooksman et al. (28) generated a transgenic model in which yellow fluorescent protein (YFP) was inserted into exon 2 of the *Prdm1* gene contained on an exogenous bacterial artificial chromosome resulting in *Prdm1*-YFP mice which retained the endogenous *Prdm1* gene structure. Using this tool in combination with various cell type-specific diphtheria toxin receptor (DTR) models, the authors observed that myeloid populations differentially regulated ASC abundance in the context of an OVA prime-boost immunization model. In this instance, partial ablation of macrophages and monocytes using diphtheria toxin (DT)-treated *Ccr2*-DTR mice lead to increased Ki-67 expression by YFP^+^ ASCs and elevated percentages of ASCs within the spleen and lymph node (28). Aside from the application of this model to identify ASCs in normal situations or well-defined tissues (21, 29–31), this model has demonstrated significant utility in its ability to facilitate ASC imaging studies or the investigation of ASCs in scenarios where canonical surface markers may not apply. For example, the *Prdm1*-YFP mouse has been a key resource for the Fooksman lab as they routinely utilize intravital imaging to examine ASC dynamics within the bone marrow (29, 32), a known niche supporting ASC longevity. This has led to key findings indicating that bone marrow ASCs cluster, possess reduced motility and have altered surface phenotypes in the aged bone marrow environment (29, 32) thus indicating that changes in ASC production rate may not be the only contributing factor that leads to the accumulation of these cells during aging (29, 32, 33). Recent work from Rojas et al. (34) and Pecha et al. (35) has demonstrated the feasibility of using *Prdm1*-YFP mice to quantify ASC levels in the gut either through flow cytometry or imaging analysis. Pecha et al. performed well-structured experiments to demonstrate that the use of digestion methods such as collagenase and liberase enzymes eliminated the ability to detect surface expression of CD138. Using the *Prdm1*-YFP model, the authors were able to design an alternative gating strategy to effectively identify ASCs within gut tissues (35). Looking outside of canonical ASC niches, Rojas et al. (34) used the model in the context of experimental autoimmune encephalomyelitis to link gut IgA^+^ plasma cell migration to the central nervous system during neuroinflammation (33) while Fitzpatrick et al. (36) used *Prdm1*-YFP mice to elegantly image the presence of ASCs within the meninges of the skull. Follow up experiments revealed that meningeal ASCs were clonally related to their gut counterparts. Critically, bortezomib-mediated depletion of meningeal ASCs led to increased mortality of mice to fungal infections (36). Finally, our lab recently used *Prdm1*-YFP reporter mice to identify ASCs within the thymus (6). This system, enabled us to show that thymic ASCs expressed canonical ASC surface markers including CD138, CD44 and CD267(TACI) while single cell RNA-sequencing revealed that thymic ASCs expressed an interferon-induced gene expression program (6) which may influence their ability to sense damage-associated molecular patterns or pathogen-associated molecular patterns via upregulation of Toll-like receptor 7. In summary, the use of constitutive fluorescent reporters has propelled our understanding of ASC biology from simple and convenient identification to being able to track these cells in diverse environments.

## 3 Inducible fluorescent reporters for ASC timestamping

A major question in the field of ASC biology revolves around what determines ASC longevity. In theory, multiple contributors likely exist including intrinsic ASC transcriptional programming, extrinsic signals from the environment as well as the types of inducing stimuli and which B cells are activated (i.e., follicular vs marginal zone vs B-1). Consequently, multiple groups have developed systems allowing for the timestamping of ASCs using inducible fluorescent reporters with many relying on *Prdm1* (**Table 2**).

**Table 2:** List and brief description of inducible fluorescent reporter and timestamping models.

Mouse model	Year	Authors	Brief description
*Jchain*-GFP-CreERT2	2020	Xu et al. (53)	GFP-CreERT2 inserted into *Jchain* intron 1 crossed with inducible tdRFP reporter to demonstrate tissue-specific ASC half-lives.
*Jchain*-CreERT2	2020	Wong et al. (54)	CRISPR/Cas9 insertion of CreERT2 into *Jchain* locus crossed with inducible tdTomato reporter to examine ASC dynamics in West Nile virus infection.
*Prdm1*-tdTomato-CreERT2 (BLTcre)	2022	Robinson et al. (37)	tdTomato-CreERT2 inserted between *Prdm1* exons 6 and 7 crossed with inducible human CD4 reporter to show ASC population dynamics following NP-KLH immunization.
*Prdm1*-CreERT2-DTR (BICREAD)	2022	Liu et al. (39)	CreERT2-DTR was inserted into the *Prdm1* locus. These animals were crossed with the Ai14 tdTomato reporter strain to generate a timestamping model.
*Prdm1*-CreERT2	2023	Koike et al. (40)	CreERT2 placed downstream of the *Prdm1* promoter crossed with inducible tdTomato reporter to examine how isotype and tissue affect ASC longevity.
*Prdm1*-tdTomato-CreERT2 (BLTcre)	2023	Robinson et al. (41)	Examined ASC longevity in the context of the aging niche and competition from newly formed ASCs.
*Jchain*-CreERT2	2024	Tellier et al. (55)	Showed that gene expression profiles of TrPC populations were regulated in part by their niches.

In 2022, Tarlinton and colleagues (37) began utilizing the *Prdm1* locus to generate mouse models to study ASC longevity and population dynamics. During this time, the Tarlinton group generated the BLTcre mouse line that possessed a tdTomato reporter and tamoxifen-inducible Cre recombinase (CreERT2) downstream of exon 6 within the *Prdm1* locus (37). In this model, tdTomato was constitutively expressed by ASCs and provided a method for identification when combined with CD138 staining. To serve as a timestamping model, the authors bred BLTcre animals with an inducible reporter line in which human CD4 was inserted downstream of a LoxP-flanked *Mcl1* gene (38). Using these animals to examine a polyclonal response against 4-hydroxy-3-nitrophenyl acetyl conjugated to keyhole limpet hemocyanin (NP-KLH), a classic T cell-dependent model antigen, it was shown that NP-specific IgG1^+^ bone marrow ASCs were steadily generated over the course of the response and that ASCs with human CD4 induced at later timepoints possessed indicators of increased affinity maturation (i.e., likely produced later within the germinal center response). However, since total NP-specific IgG1^+^ ASC numbers in the bone marrow did not increase over the course of the experiments, it was concluded that ASCs generated early in the response were likely outcompeted by their counterparts formed later. What this means for the survival of these “early” ASCs is still to be determined. That is, did these cells simply “die off” due to a lack of survival signals or in their displacement, or did these ASCs migrate to alternate tissue sights subsequently establishing long-term residency? Also in 2022, Liu et al. (39) developed what is now known as the BICREAD mouse model that incorporated not only CreERT2 into the *Prdm1* locus, but also DTR separated by a P2A linker. These animals were bred to the Ai14 tdTomato reporter strain to allow for timestamping experiments that will be discussed in depth within the following section given the reliance on the DTR aspect of the system.

In early 2023, Koike et al. (40) developed a mouse strain which incorporated a CreERT2 upstream of the *Prdm1* gene exon 1. Upon combining with the Ai14 mouse strain which possesses a floxed tdTomato expression cassette, the authors were able to acutely label ASCs as tdTomato^+^ and track the longevity of these cells. These studies demonstrated that ASC half-life differed not only between antibody isotypes within a single tissue but also between different tissues. For example, the half-lives of bone marrow ASCs were hierarchal with IgM > IgA > IgG after 1 year of tracking (40). In supplementary studies, ASCs of the same isotype were compared between different tissues showing tissue-dependent half-life such that IgM ASCs in the bone marrow had a half-life of ~178.8 days whereas the half-life of IgM ASCs in the spleen was ~46.0 days (40). This was likely due to differences in ASC composition as spleen ASCs were enriched for more immature phenotypes. By integrating a *Cd138*-DTR allele, the authors also examined the developmental relationship between difference ASC populations which is discussed in greater detail below. Later in 2023, Robinson et al. (41) again utilized BLTcre/human CD4 reporter mice to provide critical insights as to how ASC longevity can be influenced by the age of the niche as well as the influx of newly formed ASCs. Capitalizing on the knowledge that B220 is expressed by short-lived plasmablasts, the authors were able to further resolve which ASCs were likely long-lived based on the analysis of MHC II and SLAMF6 expression. Using these markers, it was concluded that longevity was greatest in MHC II^LO^ SLAMF6^LO^ ASCs followed by those with a MHC II^+^ SLAMF6^LO^ phenotype with MHC II^+^ SLAMF6^+^ cells being the least long-lived (41). Examination of the ability of newly formed ASCs to displace those that were already established suggested that the decay of preformed ASC populations occurred independently. Related to longevity, the term “inflammaging” was coined to define the chronic, low-level increase in systemic inflammation over the course of aging (42). This phenomenon is represented in part by the increase in IL-6, a cytokine known to promote the generation and/or maintenance of long-lived plasma cells (43, 44). Therefore, the evolution of ASC niches is likely continual throughout the life of an organism and would not necessarily be predicted to occur in synchrony across multiple individuals within a population. As a result, interpretations of ASC longevity experiments in the context of significant organismal aging should be taken with caution as it is difficult to draw conclusions without knowing the number of ASC niches present in an organ that can or cannot accommodate an influx of new cells.

As the desire to track ASC longevity has increased, so has the use of drivers to promote CreERT2 expression. For example, multiple models have been produced using *Jchain* as a sight of CreERT2 insertion. Since the 1970s, Jchain has been implicated in the assembly of polymeric antibodies (mainly IgA and IgM) and it has been shown to play a pivotal role in antibody transport via the polymeric immunoglobulin receptor (45–51). Further establishing *Jchain* as a suitable choice to drive CreERT2 expression; *Jchain* is thought to be highly expressed in all ASC subsets regardless of antibody isotype (52) and *Jchain* expression is minimally if not rarely expressed in other cell types when compared to ASCs (53). Capitalizing on the above in 2020, Xu et al. (53) generated a model in which *Jchain* drove expression of GFP and CreERT2 with GFP being a flow cytometric surrogate to indicate CreERT2 expression. In combination with an inducible tandem-dimer red fluorescent protein (tdRFP) reporter, the authors were able to show tamoxifen-inducible Cre activity in IgM, IgA and IgG1 ASCs (53). Similar to Koike et al. (40), the use of *Jchain*-GFP-CreERT2 mice revealed tissue-specific half-lives with bone marrow GFP^+^ tdRFP^+^ ASCs having a half-life of ~251.9 days with a half-life of ~31.6 days observed for their spleen counterparts (53). Proximal to this time, Wong et al. (54) independently generated a *Jchain*-CreERT2 animal using CRISPR/Cas9 gene editing that was crossed to the Ai14 tdTomato reporter line. In the context of West Nile virus, tdTomato labeling was observed in ASCs as expected; however, a high percentage of labeling was found in germinal center B cells (54) which differed from the Xu et al. (53) model where CreERT2 activity in germinal center B cells was rather insignificant. While the discrepancy of these results is unclear, it may be partially due to different immunizations (West Nile virus versus sheep red blood cells) or tamoxifen treatment protocols. Notably in 2024, Tellier et al. (55) used the *Jchain*-CreERT2 mice generated by Wong et al. (54) combined with the inducible tdTomato and *Prdm1*-GFP reporter systems to explore ASC longevity across various mouse tissues. The authors showed that tissue resident plasma cells (TrPCs) possessed transcriptomes that were influenced by both their induction site (i.e., site of generation) as well as their effector site (55). This blended programming may allow TrPCs to adapt to their location and survive long-term. While typical ASC populations were investigated such as those from the spleen and bone marrow, ASCs were also studied from tissues including the colon, mammary gland and adipose tissue among others to provide new insights into the growing diversity of ASCs (55). In total, the studies elaborated on above serve to demonstrate the significant role of timestamping methods to providing a growing understanding of how ASC populations evolve over time or are influenced by factors such as tissue residency. However, a not often discussed but significant caveat to the above models and in any timestamping experiment is specificity as it relates to the cell type of interest. As seen in the context of germinal center B cell labeling, the choice of CreERT2 driver, induction protocol and even the type of immunization (e.g., a bolus of sheep red blood cells versus viral infection) may lead to unintended labeling of upstream B cells that could contribute long-term to ASC production well after the cessation of timestamping. Thus, it remains critical to assess label retention (e.g., tdTomato positivity) in non-ASC B cell populations and take these results into consideration when drawing conclusions regarding ASC longevity.

## 4 Models of targeted ASC depletion

ASCs are known to regulate processes such as hematopoiesis (33, 56) and mast cell survival (57), inflammation (34, 58) as well as the evolution of the immune response (59–62). Therefore, being able to target and deplete these cells *in vivo* may provide important insights into a number of physiological and pathological processes. Multiple non-genetic approaches have been developed to accomplish the above goal such as using antibody-mediated depletion and chemical inhibitors to ablate ASCs. The laboratory of Dr. Sherrie Morrison had previously developed mouse anti-mouse CD138 antibodies for the purposes of targeting multiple myeloma (an ASC-derived cancer) cells (63). These antibodies fused the variable region from the commercially available clone 281-2 (64) to the mouse IgG2a constant region generating a reagent that would not elicit an immune response *in vivo* against rat Fc-derived peptides present in the commercial clone. Additionally, these antibodies could potentially harness Fc-mediated mechanisms to deplete ASCs beyond just impaired CD138 driven survival signals (65). Subsequently, mouse anti-mouse CD138 antibodies were used to deplete ASCs in the aging bone marrow thus demonstrating a key role for ASCs in regulating age-associated patterns of hematopoiesis (33). Studies using the standard rat anti-mouse 281–2 antibody have shown some success in depleting ASCs from the bone marrow; however, this appeared to be a result of mobilization into the peripheral blood (66, 67) rather than termination of cellular existence. As an alternative to antigen-specific targeting, chemical regulators of biological pathways important to ASC generation and/or survival have been utilized. Perhaps the most well-known of these is bortezomib which is a proteasomal inhibitor that indirectly leads to decreased NF-κB signaling via impaired degradation of IκB (68). Bortezomib has been used to deplete ASCs during viral infection (69) and in the context of autoimmune disease as demonstrated with NZB/W F1 (70, 71) and MRL-lpr (71) mice. Furthermore, this compound has been used in pre-clinical models of multiple myeloma (66). However, just like with the use of antibody-mediated depletion, utilization of chemical inhibitors is complicated by cost, dose optimization and changes in pharmacokinetics especially when organismal metabolism is altered over the course of a study. Ultimately this has led to the generation of mouse models in which ASCs can be directly targeted and depleted (**Table 3**).

**Table 3:** List and brief description of ASC depletion models.

Mouse model	Year	Authors	Brief description
*Jchain*-DTA	2001	Erlandsson et al. (47)	DT A subunit inserted into *Jchain* exon 1 to delete *Jchain*-expressing ASCs.
*Prdm1*^flox/flox^ x *Cd19*-Cre	2003	Shapiro-Shelef et al. (15)	Conditional deletion of *Prdm1* specific to the B cell lineage which cemented *Prdm1* as an ASC master transcription factor.
*Cd138*-DTR	2020	Vijay et al. (73)	DTR inserted into *Cd138* locus via CRISPR/Cas9 gene editing to allow for ASC depletion during *Plasmodium* infection.
*Prdm1*-CreERT2-DTR (BICREAD)	2022	Liu et al. (39)	Used DT-mediated ASC depletion and pulse chase methods to track new ASC formation and maturation.
*Jchain*-DTR (J-DTR)	2025	Pioli et al. (72)	DTR inserted into *Jchain* 3’ untranslated region used for DT-mediated depletion of ASCs in the bone marrow, spleen and thymus.

Engineered cell type-specific expression of DT or the DTR has become a valuable tool to ablate cells *in vivo* either constitutively or temporally, respectively. In 2001, Erlandsson et al. (47) replaced exon 1 of the *Jchain* gene with the DT A subunit coding sequence thus effectively deleting *Jchain* while also establishing a system in which *Jchain*-expressing ASCs would be deleted on a continual basis. Analysis of hybridomas and *in vivo* antibody titers suggested incomplete penetrance which may have been due to cell-to-cell expression differences or even toxin efficacy limitations. To put the timing of this model into context, it predated the generation of the *Prdm1* floxed allele which was crossed onto a *Cd19*-Cre background by Shapiro-Shelef et al. in 2003 (15). We have since built on the above to develop a mouse strain in which *Jchain* drives DTR expression in ASCs allowing for selective and temporally regulated deletion of ASCs (72). DTR expression was readily detectable in IgM, IgA and IgG ASCs from bone marrow, spleen and thymus. As expected, *Jchain*-DTR (J-DTR) mice showed highly efficient deletion of ASCs in bone marrow, spleen and thymus following DT treatment (72). Furthermore, this model allowed us to observe that ASCs are repopulated in all 3 tissues within 7 days of depletion indicating both continual and rapid replenishment of these cells at steady state (72). During the intervening time, multiple models have been generated that target DTR to different loci to drive expression in ASCs. Using CRISPR/Cas9 gene editing, Vijay et al. (73) generated *Cd138*-DTR mice in 2020 to study the outcome of ASC depletion on *Plasmodium* infection. Using this system, the authors made the key observation that robust plasmablast generation early during infection negatively impacts the immune response by essentially starving the germinal center reaction for essential nutrients. This led to the development of L-glutamine supplementation as a way to promote the germinal center response while also maintaining the early burst wave of plasmablast generation. More recently, the *Cd138*-DTR animal strain was leveraged to understand the plasmablast-to-plasma cell developmental transition and these studies definitively showed the precursor-product relationship between these 2 cell types (40). Specifically, bone marrow and spleen B220^+^ MHC II^+^ plasmablasts gave rise to B220^-^ MHC II^+^ plasma cells which subsequently generated mature B220^-^ MHC II^-^ plasma cells. These experiments provided key insights into ASC ontogeny under homeostatic conditions which can be applied in various immunization models. Finally, in 2022, Liu et al. (39) utilized the BICREAD mice to combine DT treatment with tamoxifen pulse-chase labeling to track the evolution of newly formed ASCs over time. By integrating single cell RNA-sequencing, the investigators demonstrated that long-lived IgM and IgG ASCs from spleen and bone marrow could be identified as EpCAM^HI^ whereas cells with a short-lived phenotype were CXCR3^+^. By comparison, long-lived spleen and bone marrow IgA ASCs were Ly-6A^HI^ Tigit^-^. Collectively, these data provide important insights as to the identification of long-lived ASCs which will aid in vaccine design and understanding the humoral immune response.

## 5 Discussion

Although the mouse models discussed here show the evolution of the tools to study ASC biology, it would be remiss if we did not reference the evolutionary conservation of the ASC compartment within vertebrate biology. It was discovered that over 500 million years ago, jawless (e.g., hagfish, lamprey) and jawed (e.g., teleost, mouse, human) vertebrates evolved and developed divergent but seemingly similar forms of adaptive immunity (74). While jawed vertebrates possess B and T cells that express antigen receptors constructed of variable, diversity and joining regions (i.e., VDJ), jawless vertebrates produce B cell- and T cell-like populations expressing variable lymphocyte receptors (VLRs) that are composed of flexible numbers of leucine-rich repeats (LRRs). The LRRs themselves possess significant sequence diversity leading to a theoretically diverse VLR repertoire on the order of >10^14^ in magnitude (74). Investigation of VLR gene structure and expression identified VLRB^+^ (B cell-like) (75), VLRA^+^ (αβ T cell-like) (76) and VLRC^+^ (γδ T cell-like) (77) populations. Relevant to this discussion, VLRB^+^ cells were shown to proliferate in an antigen-specific fashion and differentiate into an ASC equivalent (75) with these cells also expressing *BLIMP1* (77) and retaining surface VLRB expression (75). The latter feature is reminiscent to the maintenance of membrane immunoglobulin expression by selected ASC populations in mice (6, 33, 59, 78, 79) and humans (80–82). Therefore, it is not surprising that years of evolution have likely contributed to ASCs and their biological functions growing in complexity beyond that of solely being antibody factories. This evolutionary concept also points to the need to further understand this critical component of the adaptive immune system.

Whilst we specifically highlighted mouse models that are functionally active within ASC populations, numerous models have been developed to not only study upstream B cell development (83) but also the signaling events and extrinsic mediators required for initial ASC formation (8). Furthermore, transgenic systems have been developed over the years to study B cell tolerance (84) which is directly relevant to the formation of autoreactive ASCs. By combining these diverse models, a deeper and more comprehensive understanding of ASC biology from start to finish can be obtained. For example, the autoimmune disease systemic lupus erythematosus (SLE) is skewed towards the female sex and use of the BWF1 SLE-like mouse model has indicated increased B cell proliferation and anti-dsDNA-specific ASCs in the diseased thymus (85). By using the *Prdm1*-YFP mouse model, we were able to show increased thymic ASC production in females compared to males with thymic ASCs possessing an interferon-stimulated gene signature (6). This led to the observation that thymic B cells expressed elevated levels of Toll-like receptor 7 (6), a key molecular driver of SLE (86) among other autoimmune diseases (87). Our group is currently exploring how dysregulated regulation of thymic B cell to ASC differentiation contributes to autoimmunity. On a related topic, understanding B cell and ASC metabolism in health and disease is a topic of growing interest (88) especially considering the nutritional disparities existing throughout the world. By using mouse models to identify ways to manipulate the immune response via metabolic pathways, we may be able to design dietary strategies that are affordable and can obtain the desired immunological response whether it be in the context of a vaccine, pathogenic infection or even cancer. This concept is paralleled by the strategy previously taken to enhance the anti-*Plasmodium* response via supplementation of mice with exogenous L-glutamine (73).

A worthy point requiring further discussion revolves around one of the most pressing questions in ASC biology which is “What happens to preformed ASCs that are displaced from their survival niche by newly generated ASCs?”. This is especially pertinent when discussing durability in the context of vaccines administered for different pathogens separated over a prolonged period of time. In terms of the “big picture” question, experiments using current timestamping and labeling methods in which different vaccines are administered in staggered fashion over an extended period of time followed by pathogenic challenge with the target of the initial vaccine would indicate whether preformed ASCs are still present at sufficient amounts to provide protection. This combination of techniques may even allow for the absolute quantification of the remaining preformed ASCs. Hypothetically speaking, what would we conclude if the number of timestamped ASCs in the bone marrow was reduced following immunization with an unrelated vaccine, but pathogen protection was maintained? Maybe the displaced ASCs underwent apoptosis resulting from the absence of survival signals with the remaining ASCs being sufficient for protection. Alternatively, maybe the displaced ASCs migrated to new tissue sites which supported long-term survival and residency. To address this, incorporation of a non-ASC specific model, the photoconvertible Kaeda mouse line (89) would be advantageous. These animals have been effectively used to study ASC trafficking from the gut to the mammary glands (90) as well as hematopoietic stem cell egress from the bone marrow (91). Using this model, one would surgically expose the femurs (or other bone marrow sites), photoconvert the bone marrow cells including ASCs then track their dissemination using flow cytometry. Subsequently, ASCs can be purified and antigen specificity confirmed by ELISpot.

Expanding on this for future questions related to the nature of ASCs, an interesting concept to evaluate is the plasticity of ASC phenotypes. For example, it is appreciated that regulatory T cells (T_regs_) can adopt an ex-T_reg_ phenotype as a result of FoxP3 downregulation in disease settings and become pathogenic in nature (92). This was elegantly demonstrated using *Foxp3*-Cre fate mapping in which cells that expressed FoxP3 at some point in their existence become permanently labeled by a fluorescent marker (92). To some degree, we already know that ASCs can selectively express transcription factors such as KLF2 that regulate migratory behavior and tissue localization (93, 94), but how are these factors regulated? Are they toggled on-and-off as needed or do these transcription factors become a permanent fixture of an ASC thus solidifying an indelible phenotype? The same could be said for various cytokines expressed by ASCs such as IL-10 (34, 58). As we consider how ASC phenotypes are regulated (e.g., age-associated ability to promote myelopoiesis), understanding the inherent plasticity of these cells may be key to designing therapies that allow us to tune ASC behavior or even rejuvenate “old” or dysfunctional versions of these cells. Complicating the matter is the potential of upstream ASC precursors (e.g., B cells) expressing the factors being investigated which could lead to premature labeling and inaccurate conclusions regarding ASC expression. The solution to this issue could be the generation of new fate mapping mouse models that can be activated only in the context of *Jchain* or *Prdm1* expression. Theoretically, a *Jchain*-flippase allele could be used to induce expression of an *Il10*-GFP-Cre which is preceded by a stop codon flanked by FRT sites. By integrating an inducible tdTomato reporter it would then be possible to permanently label any ASC that expressed IL-10 at some point in its existence whereas GFP expression would indicate real-time IL-10 expression. To the best of our knowledge, this system has yet to be developed and employed in ASCs; however, the idea provides a conceptual framework as to understanding the stability of ASC functional phenotypes.

While these authors have a firm appreciation of animal models, it is important to note that no model is perfect as first recognized by George E. Box, a British statistician. While Box’s insights pertained to statistics and mathematical modeling (95), the commentary is equally relevant when considering biological systems. In the context of ASC biology, some *Jchain*-driven models may possess off target effects specific to a subset of germinal center B cells (54). Similarly, *Prdm1* gene expression is heightened in ASCs but in no way is *Prdm1* expression exclusive to this immune cell type (96). Ultimately, this may necessitate the need to continue the quest for ASCs models that rely upon genes truly specific to the ASC lineage. Alternatively, it may be possible to design hypomorphic versions of *Prdm1* or *Jchain*-driven models or those driven by other gene loci that can increase the therapeutic window (e.g., specificity of CreERT2 induced labeling) by taking advantage of expression differences between ASCs and other relevant immune cell types. This may be aided by examining previously existing studies such as comparing transcriptional signatures identified for TrPCs (55) versus those that correlate with B cell populations sitting immediately upstream of ASC differentiation (97). Until then, the best strategy especially in the context of genetic knockouts will be to utilize multiple distinct models and look for overlapping and agreeable phenotypes depending on the topic being explored. Furthermore, it will be important to examine the mouse models of choice to determine if any off-target effects exist which can be mitigated.

As we continue to develop new strategies to study ASC biology in animal models, it is important to be cognizant of how these results relate to the human condition. Clearly, some observations in mouse models have been translated to humans such as downregulation of CD19 and Ki-67 being indicative of presumed longevity and maturation (81, 98), respectively. Furthermore, the advent of single cell RNA-sequencing technologies has allowed the field to decipher ASC heterogeneity in mice and humans while finding further areas of common ground bridging observations from mice to humans. One highlight of this is a recent study that examined IgE ASC populations in a mouse model of chronic allergen exposure (99). Overall, mouse IgE ASCs were characterized by gene expression programs related to survival and endoplasmic reticulum stress that were enhanced related to other isotypes. These results were recapitulated when examining ASC populations from the bone marrow of allergic human donors (e.g., cat allergies, shrimp allergies, etc.) (99). However, the ability to correlate ASC studies in animals to that of those in humans still faces some challenges such as species-specific differences in cell surface markers. Perhaps the best example of this is CD138 which is found on essentially all mouse ASCs including plasmablast and plasma cell populations (5–7, 10). In contrast, work from Lee and colleagues has shown CD138 expression to be restricted to the most mature of human ASCs (81, 100) indicating that isolation protocols may skew the ability to perform equivalent comparisons between mouse and human ASCs. Furthermore, the environments in which animal studies are conducted can alter outcomes and quite frankly be representative on a micro-scale of the type of ASC heterogeneity that exposure to the environment (e.g., pollutants, microbes, etc.) can induce in human populations. This was illustrated by Wilmore et al. (101) who showed that C57BL/6J mice housed in different specific pathogen-free environments at The Jackson Laboratory and the University of Pennsylvania possessed variations in microbiomes which contributed to altered production of IgA ASCs found in the bone marrow. In this sense, moving towards a more natural setting similar to Wayne Potts and the “mouse barn” (102) may provide the opportunity to examine ASC development and function in a more realistic and human-relatable environment.

In summary, the last 20 plus years has seen significant advancement of ASC focused animal models ranging from constitutive reporters to strategies for *in vivo* depletion. This has led to the filling of many knowledge gaps regarding ASC generation, diversity and longevity as it pertains to different forms of stimuli, responding B cell populations as well as tissues throughout an organism. Without a doubt, these findings have been truly significant; however, we still have a long road ahead before we obtain a complete picture as to how ASCs fully contribute to immunity and host physiology in health and disease especially as it relates to human biology.
